# NapB in excess inhibits growth of *Shewanella oneidensis* by dissipating electrons of the quinol pool

**DOI:** 10.1038/srep37456

**Published:** 2016-11-18

**Authors:** Miao Jin, Qianyun Zhang, Yijuan Sun, Haichun Gao

**Affiliations:** 1 Institute of Microbiology and College of Life Sciences, Zhejiang University, Hangzhou, Zhejiang, 310058, China

## Abstract

*Shewanella*, a group of ubiquitous bacteria renowned for respiratory versatility, thrive in environments where various electron acceptors (EAs) of different chemical and physiological characteristics coexist. Despite being extensively studied, we still know surprisingly little about strategies by which multiple EAs and their interaction define ecophysiology of these bacteria. Previously, we showed that nitrite inhibits growth of the genus representative *Shewanella oneidensis* on fumarate and presumably some other CymA (quinol dehydrogenase)-dependent EAs by reducing cAMP production, which in turn leads to lowered expression of nitrite and fumarate reductases. In this study, we demonstrated that inhibition of fumarate growth by nitrite is also attributable to overproduction of NapB, the cytochrome *c* subunit of nitrate reductase. Further investigations revealed that excessive NapB *per se* inhibits growth on all EAs tested, including oxygen. When overproduced, NapB acts as an electron shuttle to dissipate electrons of the quinol pool, likely to extracellullar EAs, because the Mtr system, the major electron transport pathway for extracellular electron transport, is implicated. The study not only sheds light on mechanisms by which certain EAs, especially toxic ones, impact the bacterial ecophysiology, but also provides new insights into how electron shuttle *c*-type cytochromes regulate multi-branched respiratory networks.

*Shewanella*, a group of ubiquitous γ-proteobacteria, exhibit a remarkable versatility in respiration, allowing the use of a diverse array of electron acceptors (EAs), including fumarate, nitrate, nitrite, thiosulfate, trimethylamine *N*-oxide (TMAO), dimethylsulfoxide (DMSO), Fe(III), Mn(III) and (IV), Cr(VI), U(VI), and so forth^1^. This feature confers the bacteria great potential in bioremediation of heavy metals and energy generation via microbial fuel cells^2^,^3^. Moreover, because many physiological traits are either not found in or distinct from well-characterized model microorganisms such as *Escherichia coli*, the genus representative, *Shewanella oneidensis* is now emerging as a research model for general bacterial physiology^4^,^5^. As the signature of the genus, respiratory versatility has been extensively studied and a number of respiratory pathways have been elucidated over the last two decades^6^,^7^,^8^,^9^,^10^,^11^,^12^. However, in environments where *Shewanella* thrive it is common that multiple EAs are present, but up to date, how such a situation, especially with toxic EAs, defines ecophysiology of these bacteria remains largely elusive.

Nitrite, which is ubiquitous and highly toxic, is such an EA. To respire nitrite, *S. oneidensis* uses atypical periplasmic nitrite reduction (NRF) system, featured by the lack of the NrfH or NrfBCD complex, which in many bacteria are essential and dedicated quinol dehydrogenases to reductase NrfA^10^,^13^,^14^. CymA, a cytoplasmic-membrane-bound cytochrome *c*, is recruited to complete the system in *S. oneidensis*^10^,^15^. CymA in fact functions as an electron transport hub, connecting the quinol pool to a number of reductases, such as those for nitrate, fumarate, DMSO, and metals, either directly or via electron shuttles such as CctA and ScyA^16^,^17^. Conceivably, there would be preferential electron acceptors for CymA. Indeed, a case has been reported. CymA favors NapB, the small subunit of periplasmic nitrate reductase (NAP) which transports electrons to NapA (the large subunit of NAP), preventing nitrate and nitrite respiration from occurring simultaneously^10^. In the absence of NapB, CymA delivers electrons to both NapA and NrfA, enabling nitrate and nitrite to be respired at the same time.

Nitrite has been traditionally used as preservative in meat products to inhibit growth of bacterial pathogens for centuries. The antimicrobial action of the nitrite moiety is through either interfering with protein cofactors, such as Fe-S clusters, heme, and lipoamide, or promoting the formation of reactive nitrogen species^18^. Although nitrite has been reported to be able to directly react with the ferric heme, forming an intermediate species with ferrous-nitrogen dioxide character^19^,^20^, it is widely accepted that the molecule exerts antimicrobial activity largely via nitric oxide (NO), which can be generated from nitrite spontaneously in highly acidic or reducing environments or enzymatically^21^,^22^.

However, this is not the case in *S. oneidensis* given that nitrite is similarly effective in a NO-free environment^23^,^24^. During aerobic growth of *S. oneidensis*, the primary target of nitrite is the cytochrome *cbb*_3_ oxidase, the enzyme complex predominantly responsible for oxygen respiration^25^,^26^,^27^,^28^. When non-oxygen EAs are used to support growth, nitrite inhibits respiration of CymA-dependent but not -independent EAs^24^. This is achieved via repression of cyclic adenosine monophosphate (cAMP) production, a second messenger required for activation of cAMP-receptor protein (Crp). While as a global regulator Crp-cAMP mediates transcription of a large number of genes^25^,^26^,^29^,^30^,^31^, it plays a particularly primary role in regulation of CymA-dependent respiration^32^,^33^. In the case of fumarate, if nitrite is not promptly removed, intracellular cAMP levels drop; this impairs Crp activity, leading to substantially reduced production of both nitrite reductase and fumarate reductase FccA. In contrast, nitrite can be simultaneously respired with CymA-independent EAs, such as TMAO, resulting in enhanced biomass.

In this study, we continued our effort to unravel mechanisms underlying nitrite inhibition of non-oxygen EA respiration. By using a transposon-based random mutagenesis, we identified that the *napB* gene is associated with growth inhibition by nitrite. Growth inhibition is in fact due to excessive NapB induced by nitrite. Further investigations revealed that NapB in excess inhibits respiration of all EAs tested, including oxygen. This is seemingly due to that NapB dissipates electrons from the quinol pool, presumably to some extracellular EAs, resulting in an electron shortage for growth-supporting EAs. Additionally, we showed that NrfA production is linked to NapB levels, which relies on both Crp-cAMP and NarQP regulatory systems for nitrate/nitrite sensing and respiration.

## Methods

### Bacterial strains, plasmids and culture conditions

All bacterial strains and plasmids used in this study were listed in Table 1. Information for primers used in this study was available upon request. For genetic manipulation, *E. coli* and *S. oneidensis* were grown in Lysogeny broth (LB, Difco, Detroit, MI) under aerobic conditions at 37 and 30 °C, respectively. When appropriate, the growth medium was supplemented with chemicals at the following final concentrations: 2,6-diaminopimelic acid (DAP), 0.3 mM; ampicillin, 50 μg/ml; kanamycin, 50 μg/ml; gentamycin, 15 μg/ml; and streptomycin, 100 μg/ml.

Growth of *S. oneidensis* strains under aerobic or anaerobic conditions was determined by recording the optical density of cultures at 600 nm (OD_600_). M1 defined medium containing 0.02% (w/v) of vitamin free Casamino Acids and 30 mM lactate as electron donor was used as previously described^34^. For aerobic growth, mid-log cultures were inoculated into fresh medium to an OD_600_ of ∼0.05 and shaken at 200 rpm at 30 °C. For anaerobic growth, cultures were purged with nitrogen and inoculated into fresh media prepared anaerobically to an OD_600_ of ∼0.05. EAs used in this study included nitrite (2 mM), fumarate (20 mM), TMAO (20 mM), and goethite (10 mM).

### In-frame mutant construction and complementation

In-frame deletion strains for *S. oneidensis* were constructed using the *att*-based fusion PCR method as described previously^35^. In brief, two fragments flanking gene of interest were amplified by PCR, which were linked by the second round of PCR. The fusion fragments were introduced into plasmid pHGM01 by using Gateway BP clonase II enzyme mix (Invitrogen) according to the manufacturer’s instruction. Verified mutagenesis vectors were maintained in *E. coli* WM3064, which was used as the donor for subsequent conjugation, resulting in vector transfer into *S. oneidensis*. Integration of the mutagenesis constructs into the chromosome was selected by resistance to gentamycin and confirmed by PCR. Verified transconjugants were grown in LB broth in the absence of NaCl and plated on LB supplemented with 10% sucrose. Gentamycin-sensitive and sucrose-resistant colonies were screened by PCR for deletion of the target gene. Mutants were verified by sequencing the site for intended mutation.

The majority of mutants used in this study are previously constructed and verified by genetic complementation (Table 1). For newly constructed mutants, plasmid pHG102 was used in genetic complementation of mutants^36^. The coding sequence of the target genes was amplified and inserted into multiple cloning site of pHG102 under the control of the *S. oneidensis arcA* promoter, which is constitutively active^37^. For inducible gene expression, gene of interest generated by PCR was introduced into pHGE-P*tac* under the control IPTG-inducible promoter P_*tac*_^38^. After sequencing verification, resulting vectors were transferred into the relevant strains via conjugation for complementation and/or expression.

### Transposon mutagenesis

A random mutation library for the *S. oneidensis* wild-type was constructed with pHGT01, which is a transposon vector with a strong promoter embedded in the transposable region^28^. Nitrite resistance suppressor strains were selected with gentamycin and 2 mM nitrite. According to the number of colonies on the control plates (Gm^+^Nitrite^−^), the transposon library was estimated to contain more than ∼20,000 individual insertion mutants. Colonies, obtained from Gm^+^Nitrite^+^ plates, were subjected to the mapping of the transposon insertion sites by using the arbitrary PCR^39^.

### Chemical assays

Mid-log phase cultures were used to inoculate Bis-tris propane (BTP) buffer (pH 5.8) with goethite as sole EA to ∼0.1 of OD_600_ and incubated under anaerobic condition. Concentrations of Fe (II) and nitrite of cultures were quantified using ferrozine reagent and a modified Griess assay spectrophotometrically at 562 nm and 540 nm, respectively^40^,^41^. Standard curves were made using ferrous sulphate dissolved in 0.5 mol/L hydrochloric acid.

### Nitrite sensitivity assay

Cells of *S. oneidensis* strains grown to the late-log phase were adjusted to approximately 10^7^ CFUs/ml, followed by 10-fold serial dilutions. Ten microliter of each dilution was spotted onto plates containing nitrite of varying concentrations. Nitrite-free plates were included as control. The plates were incubated at 30 °C before being read. The assays were repeated at least three times with similar results.

### Cytochrome oxidase activity assay

Visual analysis of the *cbb*_3_-HCO activity was done by staining colonies with the agents for the Nadi Assay. Nadi reactions were carried out by the addition of α-naphthol and *N*′*,N*′-dimethyl-p-phenylenediamine (DMPD) on LB agar plates^42^. Colonies were timed for formation of the indophenol blue.

### Promoter activity assay

The activity of promoters of interest was assessed using a single-copy integrative *lacZ* reporter system as described previously^14^. A fragment covering the sequence upstream of each operon tested from −300 to +1 was then amplified and cloned into the reporter vector pHGEI01, verified by sequencing, and the correct plasmid was then transferred into relevant *S. oneidensis* strains by conjugation. Once transferred into *S. oneidensis* strains, pHGEI01 containing promoter of interest integrates into the chromosome and the antibiotic marker is then removed by an established approach^25^. Cells grown to the mid-exponential phase under experimental settings were collected and β-galactosidase activity was performed with an assay kit as described previously^36^.

### SDS-PAGE, heme-staining, and Immunoblotting assays

Unless otherwise noted, cells of the late exponential phase were harvested, washed with phosphate buffered saline (PBS), resuspended in the same buffer, and sonicated. Protein concentrations of the cell lysates was determined by the bicinchoninic acid assay (Pierce Chemical). For heme-staining, the cell lysates were separated on SDS-PAGE using 12% polyacrylamide gels and stained with 3,3′,5,5′-tetramethylbenzidine (TMBZ) as described elsewhere^43^. Immunoblotting analysis was performed essentially the same as previously described^44^. Proteins separated on SDS-PAGE were electrophoretically transferred to a polyvinylidene difluoride (PVDF) membrane according to the manufacturer’s instructions (Bio-Rad). The gels were blotted for 2 h at 60 V using a Criterion blotter (Bio-Rad). The blotting membrane was probed with rabbit polyclonal antibodies against NrfA. The goat anti-rabbit IgG-HRP (horseradish peroxidase) (Roche Diagnostics) was used as the secondary antibody (1:5,000) and the signal was detected using a chemiluminescence Western blotting kit (Roche Diagnostics) in accordance with the manufacturer’s instructions. Images were visualized with a UVP imaging system.

#### Identification of transcriptional start sites

*S. oneidensis* cells were grown in medium with required additives (either 5 mM nitrate or 2 mM nitrite for induction) to the mid-log phase, collected by centrifugation, and applied to RNA extraction using the RNeasy minikit (Qiagen, Shanghai) as described before^27^. RNA was quantified by using a NanoVue spectrophotometer (GE healthcare). The transcriptional start sites of *acpP* and *fabF1* were determined using Rapid Amplification of cDNA Ends (RACE) according to the manufacturer’s instruction (Invitrogen, Shanghai) as recently used^30^. In brief, reverse transcription was conducted on preprocessed RNA without 5′-phosphates followed by nested PCR suing two rounds of PCR reactions. PCR products were applied to agarose gel separation, purification of the 5′-RACE products, and inserted into the pMD19-T vector (Takara, Dalian) for direct DNA sequencing. The first DNA base adjacent to the 5′-RACE adaptor was regarded as the transcription start site.

### Other analyses

To estimate relative abundance of proteins in gels, the intensities of bands were quantified using ImageJ software^45^. Student’s *t* test was performed for pairwise comparisons. In figures, values are presented as means +/− standard deviation (SD).

## Results

### Screening for suppressors of nitrite inhibition in *S. oneidensis*

Nitrite inhibition of growth with fumarate is due to lowered intracellular cAMP levels in *S. oneidensis*^24^. As multiple factors are involved in cAMP homeostasis^31^,^33^, we reasoned that at least some in their altered abundance or absence may suppress nitrite inhibition. In attempts to identify such factors, a random mutation library was constructed from the wild-type strain using pHGT01, a mariner-based transposon vector derived from pFAC^28^,^46^. This vector has been not only used for construction of transposon insertion libraries but also applicable for cryptic operon screening because of a robust promoter embedded in the transposable sequence^28^,^47^.

**Figure 1:**
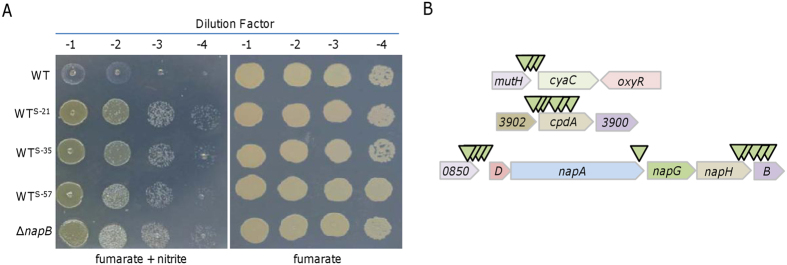
Transposon screens and terminal phenotypes. (**A**) Suppressors for nitrite inhibition of growth on fumarate. Shown were three representatives of 19 stable suppressor strains derived from the wild-type (WT): WT^S-21^, WT^S-35^, and WT^S-57^ had insertions within the *cyaC* promoter region, the *cpdA* gene, and the *napB* gene, respectively (refer to B). Densities of mid-log phase cultures were subject to 10-fold serial dilution, and 5 μl of each dilution was spotted onto plates containing 2 mM nitrite with 20 mM fumarate as EA for growth. Experiments were repeated at least three times and similar results were obtained. (**B**) Schematics indicating the approximate locations of the transposon insertions. Arrows represent transposon insertion points.

We screened a total of ∼20,000 colonies (estimated on nitrite-free control plates) on plates containing fumarate and nitrite under anaerobic conditions, and obtained 72 colonies whose nitrite inhibition is suppressed. Among them, 53 were found to be unstable with respect to resistance to nitrite (data not shown), which varied in experiments from one round to another. In addition, the insertion sites in some (16 tested) of these suppressors were either not identified successfully or within genes encoding a variety of proteins not implicated in respiration. The remaining 19 displayed much elevated resistance to nitrite consistently (Fig. 1A). Three were found to have transposon insertions in the *cyaC* promoter region (Fig. 1B). Given that the *cyaC* mutant is hypersensitive to nitrite^24^,^25^,^31^, this observation suggests that the *cyaC* gene is overexpressed because of the transposon-borne promoter. In parallel, 6 suppressors had transposons that mapped within the promoter and coding sequence of the *cpdA* gene (Fig. 1B), which encodes an enzyme dictating cAMP degradation; the *cpdA* mutant had substantially elevated cAMP levels^31^. These results are expected because nitrite inhibition can be relieved by increased cAMP intracellular levels, either by altering production/degradation or by adding the molecules exogenously^24^.

Strikingly, the remaining 10 suppressors had transposons that mapped within the *napDAGHB* region, which encodes periplasmic nitrate reductase and accessory proteins (Fig. 1B). For nitrate respiration, out of the NapDAGHB proteins only NapA is completely required as CymA is in place of otherwise essential NapC, which transports electrons from the quinol pool to NapA, via NapB if it is present^10^,^13^. The majority of Insertions were within either the *nap* promoter region or the *napB* gene and its 5′ proximity, implying a possibility that the *napB* gene is the target. Given that insertions within the coding sequence most likely destroy the function, we proposed that the NapB loss is likely to suppress nitrite inhibition of growth on fumarate.

### Nitrite inhibition is closely associated with NapB

**Figure 2:**
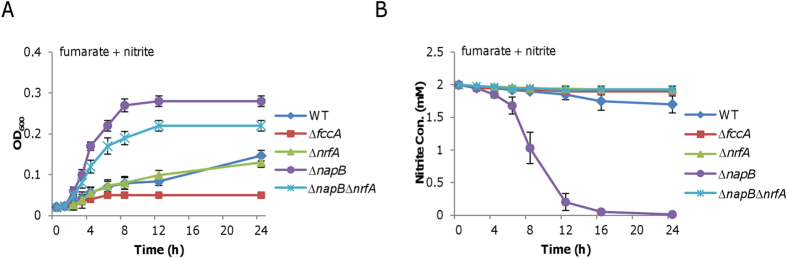
Impacts of loss of NapB. (**A**) Growth of WT, ∆*fccA*, ∆*nrfA*, ∆*napB* and ∆*napB*∆*nrfA* on 20 mM fumarate in the presence of 2 mM nitrite. The nitrite-associated defect in growth on fumarate was rescued by the loss of NapB (∆*napB*). Inability to reduce nitrite had no significant impact on growth (∆*nrfA* vs. WT). (**B**) Nitrite reduction of WT, ∆*fccA*, ∆*nrfA*, ∆*napB* and ∆*napB*∆*nrfA*. All experiments were performed at least three times with standard deviations presented as error bars.

To confirm the involvement of NapB in growth inhibition of nitrite on fumarate, we monitored growth of a ∆*napB* strain on fumarate with or without nitrite. There was no difference in growth between the wild-type and the mutant in the absence of nitrite (Fig. 1A). But when nitrite was present the Δ*napB* strain grew much better than the wild-type (Figs 1A and 2A). In addition, the capacity of reducing nitrite in the Δ*napB* strain was substantially stronger than the wild-type (Fig. 2B), implying that the improved growth is probably due to the depletion of nitrite. We then constructed a double mutant lacking both *napB* and *nrfA* genes to test this notion. Although the Δ*napB*Δ*nrfA* strain could not consume nitrite (Fig. 2B), it still exhibited growth better than the wild-type did when both fumarate and nitrite were served (Fig. 2A). In comparison, growth of an *nrfA* mutant^10^, which is deficient in nitrite respiration, was similar to that of the wild-type in the presence nitrite. Importantly, the wild-type phenotypes were restored by expression of respective *napB* and *nrfA in trans* (Fig. S1A). These results indicate that nitrite respiration has an important but not essential role in supporting growth on fumarate, implying that NapB functions as an inhibitor of nitrite reduction.

In most characterized bacterial NAP reductases, NapA and NapB, the large and small subunits respectively, are essential to nitrate respiration^13^,^48^. Although *S. oneidensis* NapB is not fully required for the process, we assessed the role of NapA in nitrite inhibition. The result revealed that a *napA* null mutant was as sensitive as the wild-type to nitrite (Fig. S1). To test whether other proteins encoded by the *napDAGHB* operon are involved in nitrite inhibition, we knocked out *napDAGH* and assessed the impact of the loss. Similar to the *napA* mutant, this deletion mutant was indistinguishable from the wild-type when cultivated on fumarate in the presence or absence of nitrite (Fig. S1B). Thus, we conclude that NapB is the only one encoded by the *napDAGHB* operon being involved in nitrite inhibition.

### Nitrite induces production of NapB

In *S. oneidensis*, the *napB* gene is the last gene in the nitrate-inducible *napDAGHB* operon^10^. We have previously shown that both *napDAGHB* and *nrfA* are regulated by the NarQ-NarP two-component system, which responds to nitrate and nitrite^10^,^44^. Apparently, the *napB* gene is expressed in cells grown on fumarate and nitrite given the significant impact of its loss; but there is no nitrate in cultures to induce the *nap* operon. In an attempt to address this dilemma, we used 5′-RACE to determine whether there is a nitrite-responsive promoter for the *napB* gene. However, no promoter in the sequence immediately upstream of the *napB* coding region was identified (data not shown). The lack of an internal promoter for *napB* was also supported by using the *lacZ* reporter (Fig. S2).

**Figure 3:**
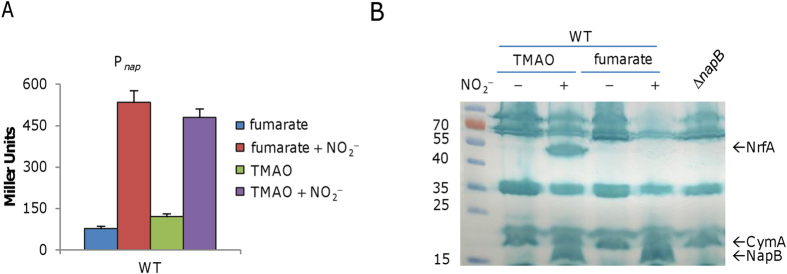
Effect of nitrite on expression of the *nap* operon. (**A**) Promoter activity measurement of P_*nap*_ by an integrated *lacZ* reporter in cells grown on fumarate, fumarate + NO_2_^−^, TMAO (20 mM), and TMAO + NO_2_^−^. Cells of mid-log phase cultures grown on indicated EAs were pelletted, processed, and subjected to β-galactosidase activity assay as described in Methods. All experiments were performed in triplicate and error bars indicate standard error. (**B**) Heme-staining results of samples used in (**A**). Samples were processed, protein contents were quantified, and the equal amounts of proteins were separated in SDS-PAGE, and then subjected to heme-staining. Note that in the presence of nitrite cells grown on fumarate but not TMAO produced extremely low level of NrfA. The ∆*napB* strain grown on oxygen was used as the control.

The operon promoter (P_*nap*_) activity was then analyzed. In the wild-type grown on fumarate, nitrite elicited P_*nap*_ induction of at least 5-fold (Fig. 3A). When CymA-independent TMAO was used as EA to support growth (not subjected to nitrite inhibition)^24^, a similar result was obtained. To confirm this, we compared cytochrome *c* profiles in cells grown on fumarate and TMAO with or without nitrite (Fig. 3B). In agreement with our previous data^24^, a band corresponding to NrfA was present in cells grown on TMAO with nitrite but barely visible in those grown on fumarate with nitrite. Evidently, a cytochrome *c* of ∼16 kD in size was present in a large amount in the presence of nitrite but regardless of EAs used to support growth. By compared to the ∆*napB* strain, we predicted that the protein is NapB. This was then confirmed by mass spectrometry (MS) analysis of the ∼16 kD band. It is worth noting that CymA was produced at relatively constant levels under all test conditions as previously reported^10^. All these data, collectively, manifest that NapB is subjected to induction of nitrite.

### NapB in excess inhibits growth

**Figure 4:**
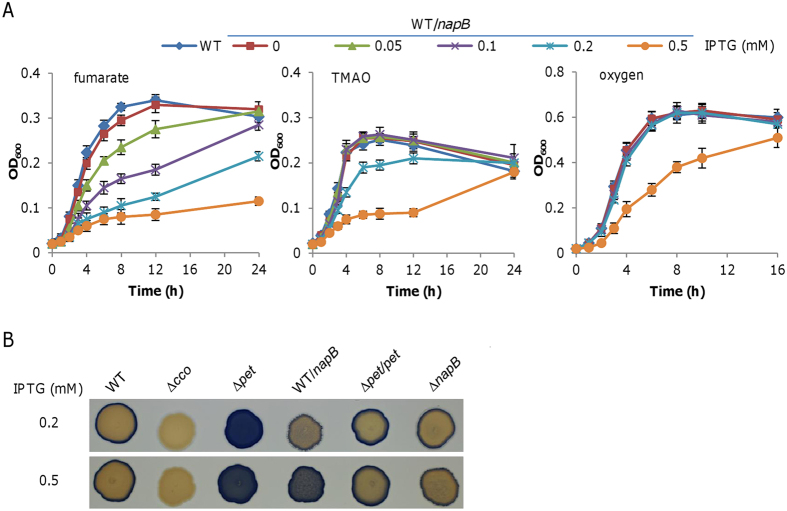
Effects of overproduced NapB on respiration of various EAs in *S. oneidensis*. In the wild-type (WT), expression of the *napB* gene was driven by IPTG-inducible P_*tac*_ within pHGE-Ptac by IPTG at indicated concentrations (mM). (**A**) Effects of overproduced NapB on growth supported by fumarate (left panel), TMAO (middle panel), and oxygen (right panel) as sole EA. All experiments were performed at least three times and error bars indicate standard error. (**B**) Effects of overproduced NapB on activities of the cytochrome *c* oxidase by the Nadi assay. The method is based on the rapid formation of indophenol blue from colorless a-naphtol catalyzed by cytochrome *c* oxidase, using *N*′,*N*′-dimethyl-p-phenylenediamine monohydrochloride as an exogenous electron donor. On plates containing IPTG at indicated concentrations, Nadi-positive and -negative strains were photographed 5 min after the reaction began. WT and previously verified ∆*cco* served as positive and negative controls. The *pet* mutant and its complemented strain, along with ∆*napB*, were included for comparison. Strains without overproducing NapB contained the empty vector. Shown were representative results from multiple experiments.

Our results thus far illustrate that NapB is highly produced in the presence of nitrite. To investigate whether nitrite inhibition of growth on fumarate, at least in part, is due to NapB in overabundance, we assessed effect of NapB in varying amounts on respiration of fumarate in the absence of nitrite. The *napB* gene was expressed in the wild-type strain under the control of IPTG-inducible promoter P_*tac*_, which is slightly leaky^49^,^50^. Overexpression of the *napB* gene led to severe growth defect on fumarate (Fig. 4A). The inhibitory effect was evident with IPTG of 0.05 mM and growth was hardly visible with 0.5 mM. For comparison, we repeated the experiment with TMAO was sole EA. Surprisingly, NapB in excess also inhibited growth, albeit less severely (Fig. 4A). Cells grew normally with IPTG up to 0.1 mM but displayed substantially impaired growth with IPTG of 0.5 mM. To assess NapB production levels in cells induced by IPTG of various levels, total proteins were extracted, separated on SDS-PAGE, and applied to heme staining. Compared to the NapB level in the wild-type grown with nitrite, IPTG at 0.05 mM and 0.2 mM had expression of more than 5 and 20-fold (estimated by ImageJ), respectively (Fig. S3).

In bacteria having a typical NAP system (NapC as quinol dehydrogenase), NapB is essential to nitrate respiration by complexion with NapA and NapA is the preferred, if not exclusive, electron acceptor for NapB^51^. Despite this, NapB in essence is an electron shuttle protein; thus this small cytochrome *c* in excess may dissipate electrons of the quinol pool, presumably via quinol dehydrogenases. As a result, there could be an electron shortage for respective terminal reductases, such as fumarate reductase FccA for growth on fumarate and TMAO reductase TorA for growth on TMAO in above described cases, leading to impaired growth. If this holds, we reasoned that oxygen respiration would be affected by excessive NapB as the process also relies on electrons from the quinol pool. Indeed, 0.5 mM IPTG caused a severe growth defect under aerobic conditions although oxygen respiration was apparently more tolerant to NapB overdose (Fig. 4A). We supposed that the defect is due to an electron shortage to the cytochrome *cbb*_3_ oxidase. To test, we conducted Nadi assay, which specifically detects the activity of cytochrome *c* oxidase^42^. We have previously demonstrated that an electron shortage for the *cbb*_3_ oxidase leads to a hyperactive phenotype, as illustrated in a strain deficient of the cytochrome *bc*_1_ complex (Fig. 4B), which functions as a quinol dehydrogenase, mediating electron transfer from the quinol pool to the *cbb*_3_ oxidase^14^,^28^. Because the *cbb*_3_ oxidase works with electrons either from the cytochrome *bc*_1_ complex or from the Nadi reagents, when electrons are no longer delivered to the *cbb*_3_ oxidase in the absence of the cytochrome *bc*_1_ complex the enzyme exclusively reacts with the Nadi reagents, exhibiting enhanced activity. Although the NapB loss did not affect activity of the *cbb*_3_ oxidase, overproduced NapB also resulted in a hyperactive phenotype of the *cbb*_3_ oxidase, albeit not so strong as that in the *bc*_1_-deficient mutant, supporting that NapB in excess reduces the amounts of electrons to the *cbb*_3_ oxidase (Fig. 4B). To further confirm this notion, we examined influences of NapB in varying amounts on expression of the *petABC* operon (encoding the cytochrome *bc*_1_ complex) by using the integrative *lacZ*-reporter. The differences in expression levels were insignificant (Fig. S4). All of these results, collectively, indicate that NapB in excess inhibits respiration of many EAs, at least including oxygen, fumarate, and TMAO, possibly by dissipating electrons of the quinol pool.

### NapB impacts NrfA production significantly in a Crp- and NarQP-dependent manner

**Figure 5:**
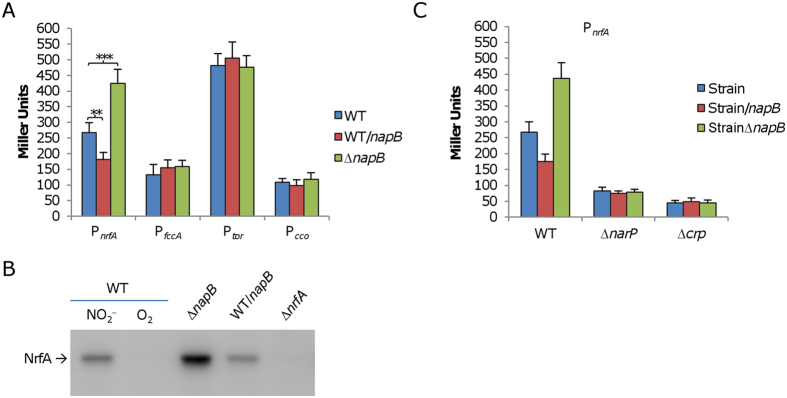
NapB at varying levels affects *nrfA* expression. (**A**) Effect of NapB at varying levels on the expression of the *nrfA, fccA, tor*, and *cco* operons in cells grown on nitrite (IPTG concentration: 0.05 mM), fumarate (IPTG concentration: 0.05 mM), TMAO (IPTG concentration: 0.2 mM), and oxygen (IPTG concentration: 0.5 mM), respectively. For each EA, the IPTG level used here was sufficiently high to induce a growth defect but could not block growth (refer to Fig. 4A). Promoter activity was assayed the same as in Fig. 3. (**B**) Results of western blotting of NrfA in nitrite-growth samples used in (**A**) WT cells grown on nitrite and oxygen were used to illustrate nitrite induction of *nrfA* expression, with ∆*nrfA* grown on oxygen as negative control. Shown was the cropped blot and the full-length gel is given in the Supplementary Information file (Fig. S6). (**C**) Dependence of NapB regulation on NarQP and Crp. Expression of the *nrfA* gene in strains lacking the NarQP system (∆*narP*) and Crp (∆*crp*) containing NapB at different levels grown on nitrite by using the integrative *lacZ* reporter as in (**A**) All experiments were performed at least three times and error bars indicate the standard error or representative results were presented.

Given that NapB in excess inhibits growth on fumarate, TMAO, and oxygen, we tested whether such inhibition is due to impaired expression of the *nrfA, fccA, tor,* and *cco* genes/operons. We overexpressed the *napB* gene in cells grown on nitrite, fumarate, TMAO, and oxygen as sole EA, respectively, and monitored expression of the genes of interest by using the integrative *lacZ* reporter system^14^. Because susceptibilities of *S. oneidensis* cells to NapB amounts with respect to growth differ significantly (Fig. 4A), we adjusted IPTG to concentrations that allow growth but at the same time elicit significant inhibition. Mid-log phase cells were collected and assayed. Clearly, in cells grown on nitrite *nrfA* expression diminished substantially, to approximately 68% relative to the control sample (the wild-type with empty vector) (Fig. 5A). In contrast, results revealed that all other tested genes were expressed in a manner independent of excessive NapB (Fig. 5A). Moreover, we repeated the experiment in the Δ*napB* strain. Consistently, in the Δ*napB* strain *nrfA* was expressed at enhanced levels but all other genes/operons, *fccA, tor,* and *cco*, were unaffected (Fig. 5A). The influence of NapB on production of NrfA in cells grown on nitrite was further confirmed by Western blot with antibodies against NrfA (Fig. 5B). All of these results manifest that NapB abundance is a critical factor governing NrfA production.

NapB resides in the periplasm and is not a DNA-binding protein, thus the protein must exert its regulatory role in an indirect manner. In *S. oneidensis*, both *nap* and *nrf* operons are controlled by the NarQ-NarP two-component system (TCS) and the Crp-cAMP complex. While NarQP TCS is an immediate regulator, Crp-cAMP functions at the higher level as a global regulatory system^44^. Based on the understanding, we predicted that NapB may rely on these systems to influence *nrfA* expression. To test this, we examined effects of NapB produced at varying levels on *nrfA* expression in relevant mutants as described above. In strains lacking either the *crp* or *narP* gene (encoding DNA-binding regulator of the TCS), the *nrfA* promoter was no longer responsive to changes in NapB quantity (Fig. 5C). Therefore, the physiological impact of NapB on *nrfA* expression depends on both Crp-cAMP and NarQP TCS.

### NapB dissipates electrons at least in part through the Mtr pathway

The presented data strongly suggest that NapB dissipates electrons of the quinol pool via CymA, TorC, and the cytochrome *bc*_1_ complex, in addition to indirectly repressing *nrfA* expression. A key issue remains unaddressed is where the electrons shuttled by NapB end given that there is no exogenous nitrate in cultures for dumping via NapA. This was further validated by the observation that nitrate was not produced endogenously to physiological relevant levels and a *napA* mutant behaved like the wild-type in the presence of overexpressed NapB (Fig. S5). As EAs whose respiration is inhibited by NapB in overabundance are inclusively located in the periplasm, we predicted that those eventually accept electrons from NapB are likely extracellularly present.

**Figure 6:**
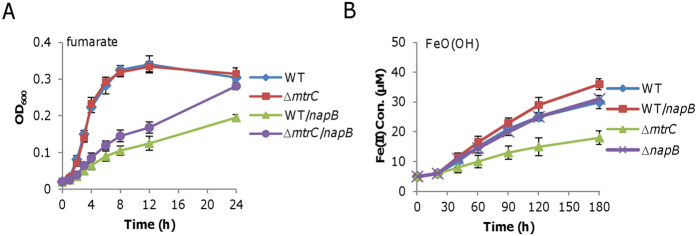
NapB in overproduction facilitates extracellular electron transfer. (**A**) Effect of overproduced NapB partially relies on the Mtr pathway. Growth of indicated strains on fumarate was measured, with NapB production induced by 0.2 mM IPTG when necessary. (**B**) Overproduced NapB promotes iron reduction. Concentrations of Fe(II) were monitored instead of growth. Production of NapB was induced by 0.2 mM IPTG. All experiments were performed at least three times and error bars indicate the standard error.

To test this, we examined the influence of the Mtr respiratory pathway on the proposed role of NapB given that Mtr dictates electron transport to extracellular EAs in *S. oneidensis*^52^,^53^,^54^. When fumarate was used as EA, a strain lacking the *mtrC* gene, encoding the major component of the Mtr pathway^55^, displayed significantly alleviated growth compared to the wild-type upon excessive NapB (Fig. 6A). This result implicates a role of the Mtr pathway in electron dissipation mediated by NapB. To provide additional evidence, we assayed effect of NapB in varying amounts on extracellular EA goethite (FeO(OH)). We monitored Fe (III) reduction of relevant strains as growth was too poor to be measured reliably. We found that NapB in overproduction significantly enhanced Fe (III) reduction compared to the wild-type without induction (Fig. 6B). However, the loss of NapB did not elicit any notable difference. Nevertheless, these results support that NapB diverts electron flux from CymA to goethite, presumably some other extracellular EAs. All together, we conclude that NapB dissipates electrons through the Mtr pathway, at least in part, in *S. oneidensis*.

## Discussion

It is well established that toxicity of nitrite to cells is largely attributed to either interfering with protein cofactors, such as Fe-S clusters, heme, and lipoamide, or promoting the formation of reactive nitrogen species^18^. As a molecule with negative charges, nitrite could not cross the inner-membrane of Gram-negative bacteria freely; specific transporters, such as *E. coli* NarK and alike, are needed to mediate uptake of nitrogen oxyanion (nitrate and nitrite)^56^,^57^. However, a large number of bacteria that are equipped with the respiratory nitrate/nitrite reductases only, including *S. oneidensis*, could not import nitrate/nitrite for the lack of such transporters. Thus, it is conceivable that some of nitrite targets may reside in the periplasm, where it is at the highest concentrations. Clearly, it is true during aerobiosis in *S. oneidensis*; the primary target of nitrite is the cytochrome *cbb*_3_ oxidase, the predominant enzyme for oxygen respiration^25^,^26^.

During anaerobiosis, however, scenarios are drastically different. In our previous study, we illustrate a mechanism for nitrite inhibition in which the molecule functions as a signal molecule rather than a toxic agent^24^. Although the exact receptor for nitrite remains elusive, the result is clear: lowered production of cAMP. In this presented study, we uncovered a parallel mechanism underlying nitrite inhibition during anaerobiosis. Although nitrite also acts as a signal molecule as in to the former, it exerts inhibition through up-regulating NapB production.

In most bacteria possessing a NAP system, NapB is a non-catalytic but essential subunit that shuttles electrons from NAP-specific quinol dehydrogenase NapC to NapA^51^. In *S. oneidensis*, NapB is not essential to nitrate reduction but improve the efficiency of NapA, conferring cells a fitness gain in nitrate-containing environments^10^. To date, there has not been any report linking NapB to other physiological processes. Nevertheless, due to its low midpoint reduction potentials, it is proposed that NapB has great thermodynamic advantage for drawing electrons from the quinol pool^58^. The data presented here definitely support the proposal. As a periplasmic cytochrome *c*, to obtain electrons from the quinol pool NapB has to interact with cytoplasmic membrane-bound quinone dehydrogenases, such as established *c*-type cytochromes NapC and CymA^15^,^51^,^58^. By overproducing NapB, we showed that this protein likely at least interacts with, in addition to CymA, the cytochrome *bc*_1_ complex and cytochrome *c* TorC, electron mediators specific for respiration of oxygen and TMAO, respectively^14^,^59^. Given that NapB also promotes Mtr-mediated respiration of extracellular EAs, it is reasonable to predict that NapB may interact with the Mtr pathway. Interestingly, although more than 40 *c*-type cytochromes are encoded in the *S. oneidensis* genome^60^,^61^,^62^, the bacterium routinely uses this strategy to increase the complexity of its respiratory pathways. For instance, periplasmic *c*-type cytochromes FccA and CctA can work with the Mtr pathway and ScyA partners with cytochrome *c* peroxidase and the *cbb*_3_ oxidase^17^,^28^,^62^.

Although the new role that NapB plays is significantly beyond being the small subunit of NAP, its coding gene is not independently transcribed but as a member of the *napDAGHB* operon. Nitrite induces expression of the *napDAGHB* operon, whose transcription is subjected to regulation of NarQ-NarP two-component system and global regulator Crp^44^. In line with this, it was revealed in this study that up-regulation of the *napDAGHB* operon by nitrite is dependent on both NarQ-P and Crp. This is somewhat unexpected because nitrite also impairs Crp activity by diminishing cAMP levels, resulting in a reduced level of overall cytochrome *c* production^24^,^37^. This is particularly true for CymA-dependent reductases, such as NrfA and FccA, whose down-regulation largely accounts for nitrite inhibition of growth on fumarate^24^. However, NapA seems exceptional. How the *napDAGHB* operon is immuned from Crp deactivation with lowered cAMP levels is unknown and worthy of further investigation. Another interesting question needed to be addressed is how NapB in excess represses expression of the *nrfA* gene. Given that NapB diverts electrons from CymA to terminal reductases rather than NrfA as presented here and before^10^, it seems that cells can sense the electron flux and regulate expression of respective reductases accordingly.

The previously undescribed mechanism by which nitrite can regulate multi-branched respiratory networks has important implications in ecophysiology. To unravel the functional mechanism of NapB, we manipulated NapB overproduction to levels sufficiently high to display inhibitory effects on growth on CymA-independent EAs, such as oxygen and TMAO. However, this would not occur in the natural environment. As a matter of fact, NapB induced by 2 mM nitrite had no inhibition on respiration of these EAs and more importantly, improved growth on TMAO, presumably some other CymA-independent EAs too, such as sulfur species^24^. By this way, nitrite conceivably provides a fitness gain when cells live in environments containing both nitrite and CymA-independent EAs. It is worth mentioning that most of *Shewanella* thrive in marine environments where TMAO and sulfur species are relatively rich. In parallel, nitrite facilitates respiration of extracellular EAs, implying that nitrite also likely confers cells an advantage in surviving and proliferating in environments that this group of EAs dominate. In *S. oneidensis*, extracellular electron transport mainly relies on the Mtr pathway, despite the existence of a variety of respiratory strategies, including direct enzymatic reduction, bacterial nanowire, or flavin shuttles^54^,^63^. We have previously shown that *S. oneidensis* prefers insoluble to soluble EAs for respiration, such as Fe(III) oxide versus oxygen^64^,^65^. By inducing NapB expression, nitrite likely contributes to this living style in the environment.

## Additional Information

**How to cite this article**: Jin, M. *et al*. NapB in excess inhibits growth of *Shewanella oneidensis* by dissipating electrons of the quinol pool. *Sci. Rep.* **6**, 37456; doi: 10.1038/srep37456 (2016).

**Publisher’s note:** Springer Nature remains neutral with regard to jurisdictional claims in published maps and institutional affiliations.

## Supplementary Material

Supporting Information

## Figures and Tables

**Table 1:** Strains and plasmids used in this study.

Strain or plasmid	Description	Reference or source
Strain		
*E. coli*		
DH5α	Host for cloning	Lab stock
WM3064	Donor strain for conjugation, ∆*dapA*	W. Metcalf, UIUC
*S. oneidensis*		
MR-1	Wild type	Lab stock
HG0608-10	∆*pet* derived from MR-1	14
HG0624	Δ*crp* derived from MR-1	37
HG0845	∆*napB* derived from MR-1	10
HG0848	∆*napA* derived from MR-1	10
HG0849-6	∆*napDAGH* derived from MR-1	This study
HG0970	∆*fccA* derived from MR-1	61
HG1778	∆*mtrC* derived from MR-1	61
HG2364-1	∆*cco* derived from MR-1	28
HG3980	∆*nrfA* derived from MR-1	10
HG3982	∆*narP* derived from MR-1	44
HG4951	∆*cymA* derived from MR-1	10
HG0845-3980	∆*napB*∆*nrfA* derived from MR-1	This study
Plasmid		
pHGM01	Ap^r^, Gm^r^, Cm^r^, *att*-based suicide vector	35
pHG102	Km^r^, promoterless broad-host vector	36
pHGC01	Integrative vector for complementation	25
pHGEI01	Integrative *E. coli lacZ* reporter vector	14
pBBR-Cre	Helper vector for antibiotic marker removal	25
pHGE-P*tac*	Km^r^, IPTG-inducible P_*tac*_ expression vector	38
pHGT01	Promoter-embedded transposon vector	28
pHGE-P*tac*-*napB*	Inducible expression of *napB*	This study
pHGEI-P*pet-lacZ*	*E. coli lacZ* under control of *pet* promoter	14
pHGEI-P*cco-lacZ*	*E. coli lacZ* under control of *cco* promoter	28
pHGEI-P*nap-lacZ*	*E. coli lacZ* under control of *nap* promoter	This study
pHGEI-P*napB-lacZ*	*E. coli lacZ* under control of *napB* promoter	This study
pHGEI-P*arcA-lacZ*	*E. coli lacZ* under control of *arcA* promoter	This study
pHGEI-P*nrfA-lacZ*	*E. coli lacZ* under control of *nrfA* promoter	This study
pHGEI-P*fccA-lacZ*	*E. coli lacZ* under control of *fccA* promoter	This study
