# Supplementary material for: NapB in excess inhibits growth of Shewanella oneidensis by dissipating electrons of the quinol pool
Source: Sci Rep. 2016 Nov 18;6:37456. doi: 10.1038/srep37456 (PMC5114592; doi:10.1038/srep37456)
Supplement: Supporting Information [file srep37456-s1.pdf]

Supporting Materials of

NapB in excess inhibits growth of *Shewanella oneidensis* by dissipating electrons of the quinol pool

Miao Jin, Qianyun Zhang, Yijuan Sun, and Haichun Gao*

Institute of Microbiology and College of Life Sciences, Zhejiang University, Hangzhou, Zhejiang, 310058, China

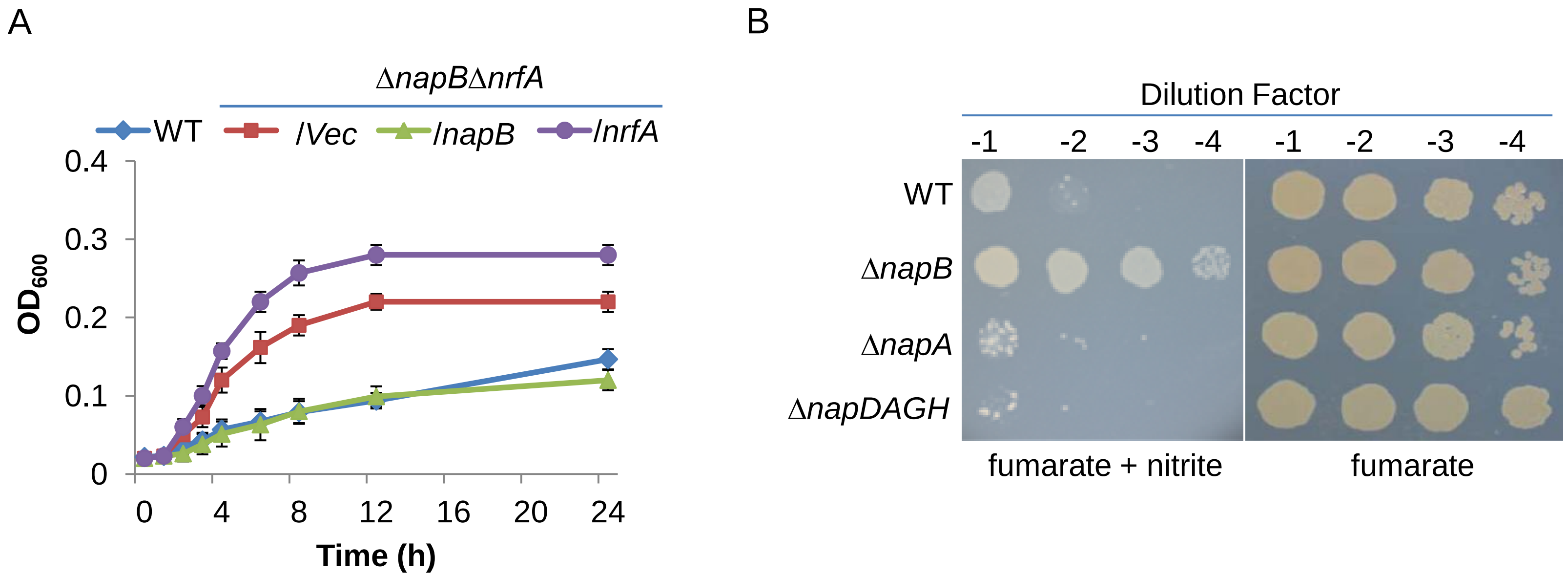

Fig. S1. A. Complementation of *nrfA* and *napB* mutation. Single mutants for *nrfA* and *napB* used in this study were confirmed by genetic complementation in a previous study (10). Vec represents empty vector. **B.** Inhibition by nitrite of growth of indicated strains on fumarate with or without 2 mM nitrite assayed as described in Fig. 1. All experiments were performed at least three times with standard deviations presented as error bars or with representative results shown.

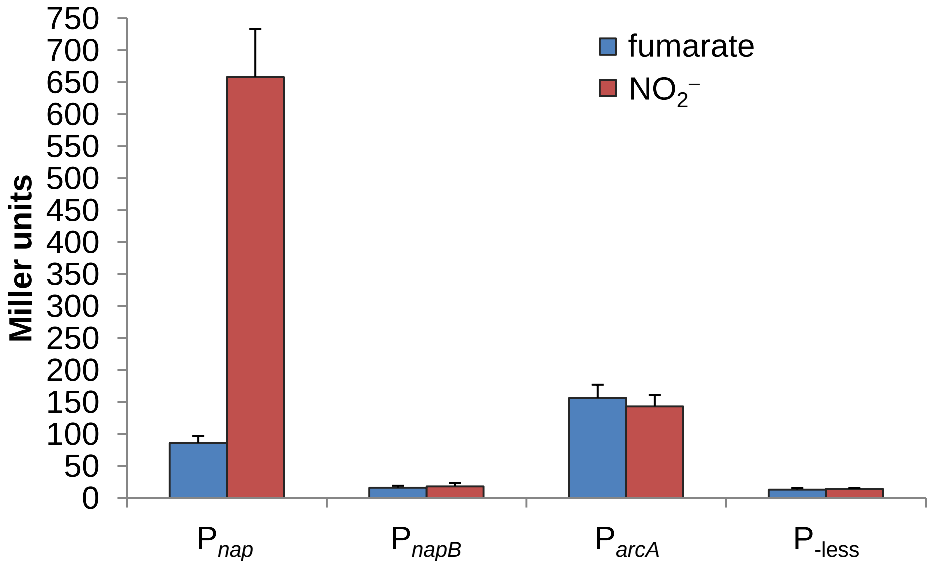

Fig. S2. The *napB* gene may be controlled by the *napDAGHB* operon promoter (P_{nap}) only. Sequences of ~300 bp upstream of the *nap* operon, the *napB* gene, and the *arcA* gene were cloned to a *lacZ*-reporter vector. Cells carrying respective vectors grown on indicated EAs to the mid-log phase were collected and subjected to β-galactosidase assay. The *arcA* promoter, relatively stable and unresponsive to EAs used (Gao et al., 2010), was used as positive control, whereas the vector carrying a copy of *lacZ* without a promoter (P_{-less}) was included as negative control. All experiments were performed at least three times with standard deviations presented as error bars.

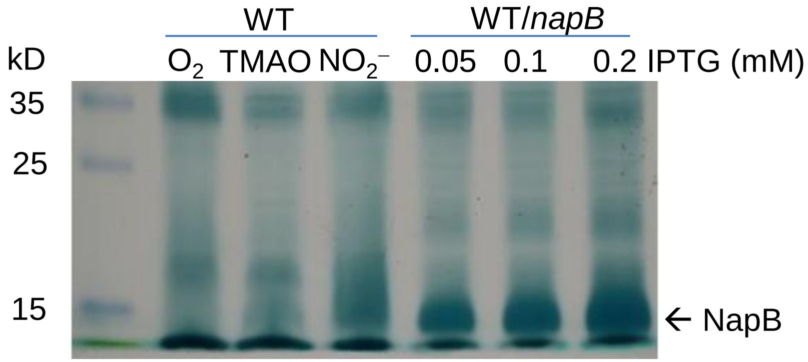

Fig. S3. Overproduction of NapB shown by heme-staining. Proteins (10 µg per lane) extracted from the wild-type (WT) without or with *napB* expressing vector strains cultivated under indicated conditions were separated on SDS-PAGE and analyzed by heme staining.

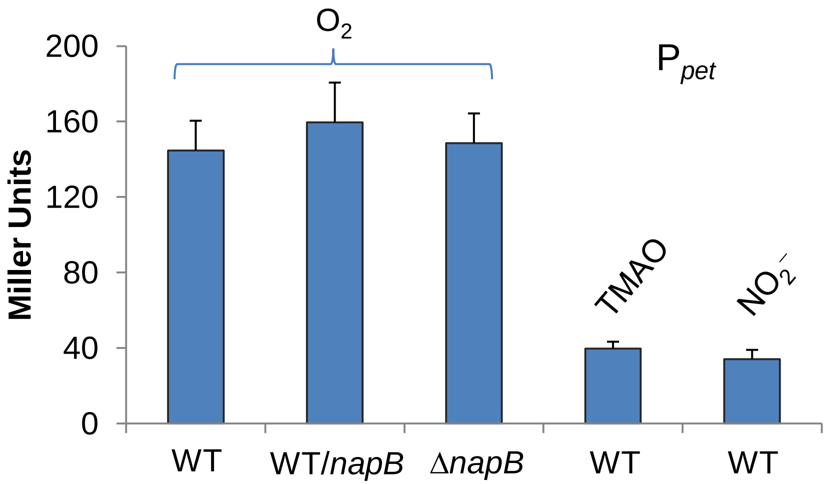

Fig. S4. NapB does not influence expression of the *pet* operon encoding the bc_1 complex. β-galactosidase driven by the *pet* promoter in a single copy in mid-exponential phase cells of indicated strains. IPTG of 0.5 mM was used to overexpress *napB*. TMAO and nitrite used as sole EA for growth were included for comparison. Data are representative of at least three independent experiments with standard deviation as the error bar.

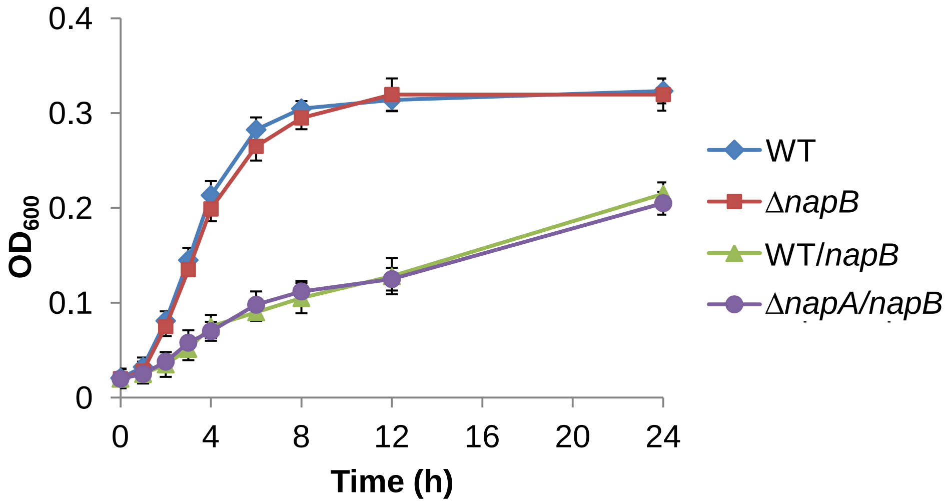

Fig. S5. NapA has no role in NapB-mediated inhibition. Growth of the *S. oneidensis* indicated strains overproducing NapB on fumarate was monitored. Expression of the *napB* gene was driven by 0.2 mM IPTG.

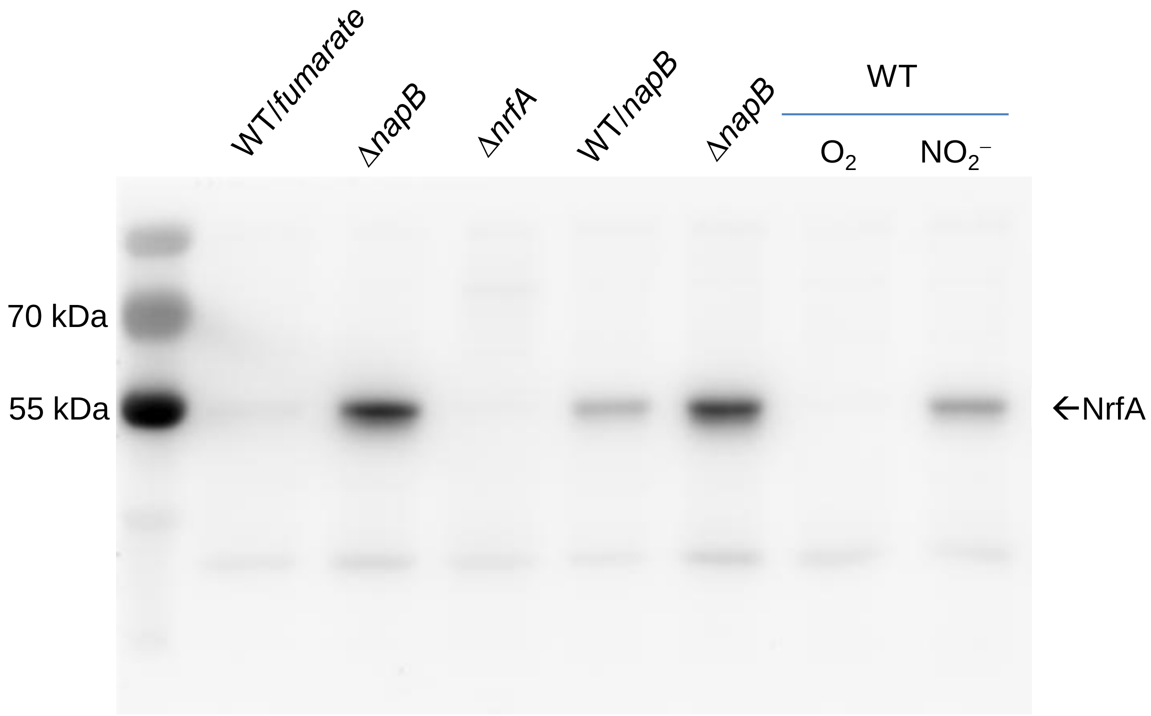

Fig. S6. Full-length gel for the cropped Western blot presented in Fig. 5B. Expression of the *napB* gene was driven by 0.2 mM IPTG.
